# So rare we need to hunt for them: reframing the ethical debate on incidental findings

**DOI:** 10.1186/s13073-015-0198-3

**Published:** 2015-07-30

**Authors:** Sebastian Schuol, Christoph Schickhardt, Stefan Wiemann, Claus R. Bartram, Klaus Tanner, Roland Eils, Benjamin Meder, Daniela Richter, Hanno Glimm, Christof von Kalle, Eva C. Winkler

**Affiliations:** EURAT Project, Department of Medical Oncology, National Center for Tumor Diseases (NCT), University Hospital Heidelberg, 69120 Heidelberg, Germany; Department of Medical Oncology, Program for Ethics and Patient Oriented Care, National Center for Tumor Diseases (NCT), University Hospital Heidelberg, 69120 Heidelberg, Germany; Department of Molecular Genome Analysis, German Research Cancer Center (DKFZ), 69120 Heidelberg, Germany; Department of Human Genetics, University Hospital Heidelberg, 69120 Heidelberg, Germany; Department of Theology, University Heidelberg, 69117 Heidelberg, Germany; Department of Theoretical Bioinformatics, German Research Cancer Center (DKFZ), 69120 Heidelberg, Germany; Department of Internal Medicine III, University Hospital Heidelberg, 69120 Heidelberg, Germany; Department of Translational Oncology, National Center for Tumor Diseases (NCT), German Research Cancer Center (DKFZ), 69120 Heidelberg, Germany

## Abstract

Incidental findings are the subject of intense ethical debate in medical genomic research. Every human genome contains a number of potentially disease-causing alterations that may be detected during comprehensive genetic analyses to investigate a specific condition. Yet available evidence shows that the frequency of incidental findings in research is much lower than expected. In this Opinion, we argue that the reason for the low level of incidental findings is that the filtering techniques and methods that are applied during the routine handling of genomic data remove these alterations. As incidental findings are systematically filtered out, it is now time to evaluate whether the ethical debate is focused on the right issues. We conclude that the key question is whether to deliberately target and search for disease-causing variations outside the indication that has originally led to the genetic analysis, for instance by using positive lists and algorithms.

## Introduction

Impressive technological advances in next-generation sequencing (NGS) have allowed an unprecedented view of the molecular bases of diseases, their development and progression. Medical research has embraced this technology, and cancer research is one of the fields at the forefront of NGS efforts. Wide-scale implementation of NGS platforms has enabled fine-grained analysis of the genome and has been accompanied by an intense debate on the ethical and legal implications of this. A very prominent matter of debate is how to handle incidental findings (IFs): it was thought that when scientists used whole-genome or exome approaches to search for the molecular basis of diseases they would not be able to avoid unintentionally finding mutations beyond their research interest associated with some of the 5000 monogenetic diseases that are currently annotated in the Online Mendelian Inheritance in Man (OMIM) database [1] or with other polygenetic conditions with health relevance.

In the 2000s, there was much concern among the scientific community about the significance of IFs in genetic and genomic research. In an influential paper by Wolf and colleagues [2], an IF was defined as “a finding concerning an individual research participant that has potential health or reproductive importance and is discovered in the course of conducting research but is beyond the aims of the study.” Bioethical discussion on IFs obtained from genomic research has mostly focused on the implications for patients, researchers and physicians. Topics that have been discussed include how best to prepare patients during the informed consent process for the potential discovery of IFs, whether researchers are obliged to report IFs to physicians or research participants, what the rights are of participants in research projects and of patients to either be informed or not to be informed about IFs, and how IFs should be assessed and classified according to their putative or established health relevance and potential benefits and burdens for participants [3].

An impressive number of articles have explored the ethical and regulatory handling of IFs [4]. Most authors start their ethical reflections assuming that genomic research will generate IFs in substantial numbers, but this premise remains hypothetical. This approach was of course legitimate to prepare for the ethical challenge of genomic research; however, it is stunning that after almost a decade of intense debate about the correct handling of IFs, most discussion still appears to be based upon hypothetical assumptions instead of empirical evidence of IFs being a common phenomenon in genomic research.

Here we propose that IFs are in fact rare, especially within genomic cancer research but also in other fields of research. We report that no IFs were found in the sequencing data obtained from various genomic research projects in Heidelberg, Germany. Referring to the wider genomic literature, we point out that there is little evidence for the occurrence of IFs in relevant numbers. We suggest that the low level of IFs is caused by the filtering approaches taken in NGS. To be clear: we do not question the existence of genetic variations with potential health relevance or the reporting obligations and responsibilities of researchers *if* they discover IFs; and we do not intend to question the related responsibilities of researchers with respect to other kinds of findings, particularly so-called research findings that occur within the intended scope of research. Instead, we intend to redirect discussion concerning IFs towards the importance and feasibility of systematically searching for such potentially disease-causing genetic variations in genetic analyses. We begin by defining the terms that we use in this article.

## Definitions of incidental and other findings

In the literature, a number of terms and definitions are used to describe IFs, such as “unanticipated findings”, “off-target results”, “ancillary information”, “secondary findings” and “additional findings”; this diversity of terms indicates the importance and difficulties of a precise definition. Here, we use the most common term (incidental findings) and understand it to mean those that (1) have a potential health or reproductive impact on the study participant, (2) are outside the scope of the research question, and (3) are not searched for actively or intentionally. It is important to distinguish IFs from “research findings”, which are health-relevant findings within the research scope, and “secondary findings”, which are health-relevant findings outside the aim of research but that are actively searched for. As an example, in a typical study of cystic fibrosis (non-cancer-related), mutations in the associated *CFTR* gene are research findings, whereas *TP53* mutations (which are cancer-related) can be either IFs, if they were not searched for, or secondary findings, if actively searched for. We do not include findings with social relevance such as misattributed paternity in any of these categories, as these do not by themselves have health or reproductive impacts and would, like most other findings outside the respective research context, only be made if actively searched for.

Some further clarifications are necessary. We draw on the distinction between research and treatment, even though we are aware that in translational contexts the distinction is sometimes blurred, and when we refer to research activities we include clinical research. We restrict the discussion to genomic research, in other words studies based on NGS techniques. And we should point out that our expertise and sequencing data are mainly in the field of cancer research. Nevertheless, we suggest that our main thesis concerning IFs should hold true for non-cancer genomic research too.

## Lack of empirical evidence for IFs being a common phenomenon

The background and initial impetus to write this paper is our own experience within the Ethical and Legal Aspects of Whole Genome Sequencing (EURAT) project group, which was set up to accompany the introduction and increasing use of NGS by biomedical research institutions in Heidelberg [5]. The EURAT project unites scientists with backgrounds in different disciplines (molecular biology, cancer genomics, human genetics, bioinformatics, law, ethics and cancer medicine) from the German Cancer Research Center (DKFZ), the National Center for Tumor Diseases (NCT), the European Molecular Biology Laboratory Heidelberg (EMBL), the Heidelberg University Medical School, Heidelberg University and the Max Planck Institute for Comparative Public Law and International Law. It aimed to address, in advance of the implementation of genome-wide sequence analysis of individuals in Heidelberg, the normative issues that are raised by NGS technologies and their application, by developing appropriate recommendations. We dedicated a considerable part of our ethical and policy recommendations to the handling of IFs, as we anticipated their occurrence on a large scale [6].

Since 2011, when sequencing was initiated, no IFs have been reported to the EURAT group. We thus started to search for proof of occurrence of IFs more systematically, and surveyed the leaders of large Heidelberg whole-genome sequencing projects about the incidence of IFs. The sequencing data have been mostly collected in cancer research projects carried out at DKFZ, NCT and EMBL, as well as in some non-cancer research projects led by the Departments of Human Genetics, Internal Medicine III and Pediatrics at Heidelberg Medical School. Together, 1429 genomes (1369 cancer, 60 non-cancer) and 2748 exomes (2567 cancer, 181 non-cancer) have been sequenced to date and no IFs have been found (unpublished data).

As the generalizability of the non-occurrence of IFs in our local data is limited, we next conducted a scoping review of the literature using a PubMed enquiry (date of analysis 31 March 2015). The first step was a search with a list of key words (connected with OR: “incidental finding” and synonyms; word families for “gene”, “genome” or “exome” and “genetic”; and “case”) in titles and abstracts for articles published in the last 10 years (538 articles). In a second step, irrelevant articles (for example, other definitions of IFs, false contexts or theoretical discussions) were excluded, and we searched in abstracts for cues for the empirical evidence of genomic IFs (38 articles). In a third step, these articles were examined in detail for empirical evidence of IFs (nine articles). The analysis of papers regarding IFs in genomics revealed that most publications focus on the ethical and legal aspects of IFs, whereas only a vanishingly small number (nine articles) address the question of their real existence. We found five case reports of single IFs [7–11]. Interestingly, the IFs mentioned in these case reports occurred in a clinical context and referred mostly to deletions identified by molecular karyotyping, which is noteworthy (see later). In the context of research, only two studies addressed the occurrence of IFs. One of them was an interview study in which 8 of 19 genomic researchers reported having encountered one or more genetic IFs over the past 12 months [12, 13]. However, as the study did not provide any definition of IFs, the interviewed researchers were referring to their personal understanding of the term, which included copy number variants with unsure clinical meaning, findings of non-paternity and findings that may not have clinical significance. The second study surveyed 234 genetic researchers in the United States. Twenty-eight (12 %) of them reported having encountered and returned IFs [14, 15]. To our knowledge this is the only empirical study that reliably suggests that IFs do occur. However, since it only notes the number of researchers who have reported IFs (or at least one IF) and does not define the number of IFs reported by the researchers or the time of the findings (within a defined period, such as a year, or during their whole professional life), the study’s informative value concerning the scale of IF occurrence remains limited. Knowing the occurrence rate of IFs would be useful for at least two reasons: first to determine whether and to what extent it is worthwhile to address potential IFs during the informed consent process, and second to anticipate and calculate the resources necessary for validating IFs and informing research participants of them when planning a research project.

Our literature search uncovered a pronounced discrepancy between the quantity and intensity of the bioethical and regulatory debate on IFs, and the scarce evidence for the occurrence of IFs at a reportable scale. However, empirical studies do exist that show a significant incidence of genetic mutations with health relevance when gene panels and positive lists are used. By “positive list” (or “minimal list”) we mean a list of genetic variant types associated with medically actionable conditions. In 2013 the American College of Medical Genetics (ACMG) published such a list of 57 genes (subsequently revised to 56) that all patients undergoing clinical sequencing should be tested for [16]. Applying this list of 56 genes, reported incidence rates of these actionable lesions vary between 0.89 % [17] and 5 % [18] of research participants. In order to investigate the pathogenicity of specific variants and to estimate their frequency in patients of European and African ancestry, Amendola and colleagues [19] analyzed exomes of 6503 research participants. Using a selected list of 112 genes, they noticed incidence rates of medically actionable findings varying between 1.1 % (African ancestry) and 2 % (European ancestry) [19]. Thus, since mutations with health relevance do indeed exist, the question is why are they not found incidentally?

## General reasons why IFs are hardly found

If we look at the wider history of clinical IFs beyond genomics, the first reported IFs were from physicians applying imaging technologies such as X-rays who discovered abnormalities beyond the initial indication. The debate on IFs gained momentum with the introduction of new computerized imaging techniques 10 years ago, when IFs were found in up to 86 % of whole-body CT scans [20]: the finer-grained and more comprehensive the imaging techniques, the higher the probability of finding IFs. Thus, the expectation of whole-genome or exome sequencing was that IFs would be frequent in genomics, similarly to the experience with pre-operative X-rays of the chest or the finding of brain lesions in MRI studies. There are a number of reasons why these expectations have not, thus far, been met.

### Genetic findings are not easily detectable

Genomic data are quite different from CT or MRI scans; the levels of depth and complexity of the information content are not the same. In contrast to imaging methods, even a trained researcher cannot “see” IFs in the genome sequence. Although the genome is made up of just four bases (if modifications are not considered), the analyzable part of the human genome comprises 3 × 10^9^ base pairs, rendering it difficult to detect any findings without substantial (bio)informatic support. The likelihood of stumbling incidentally on a point mutation is very small.

### The non-detection of IFs is a result of the methodological approach

In general terms, whole-genome sequencing is carried out in two very different contexts and with different aims. First, for cancer genome sequencing, somatic mutations within tumor cells are the prime target of the analysis. To this end, tumor and normal (blood) samples of the same individual are sequenced, and somatic mutations are identified by focusing only on the variants that differ between these two “genomes”. Using this approach, germline variants, including IFs, are excluded from detection. In some cases a small number of highly relevant genes (such as *TP53*, *BRCA1* and *BRCA2*) are investigated at the germline level (Li-Fraumeni Syndrome, familial breast cancer) as they have immediate implications for therapy and disease progression. Any other genetic conditions are currently not investigated and, thus, not detected. Second, in the analysis of genetic diseases, the genome of a research participant is usually compared with a reference genome. Based on the data of the 1000 Genomes Project, the number of variants that differ between any two genomes amounts to approximately 3 million. These differences include high numbers of normal (irrelevant) variations and relatively low numbers of disease-relevant variations. In this first step, detecting IFs is possible, but highly unlikely. The high number of alterations dictates the necessity for further filtering steps that focus the results towards the scope of the research. Since these filters are highly precise, IFs — that is, findings beyond the research scope — are technically excluded.

Interestingly, the IFs published in case reports were mostly detected through molecular karyotyping [7–10]. Since this is a rather insensitive method, only detecting major alterations at the chromosomal level, anomalies such as translocations or large rearrangements can be detected but mutations and smaller genetic abnormalities cannot. This demonstrates the critical impact of the analytical methods used for the detection of IFs. However, it might not be a coincidence that the case reports occurred in a diagnostic setting. In a study of the diagnostic usefulness of whole-exome sequencing for suspected Mendelian disorders with a wide range of phenotypes, Yang and colleagues reported 30 “IFs” among 250 patients [21]. They used filter techniques to retain 400–700 variants of potential clinical impact out of 200,000–400,000 single nucleotide variations with the reference genome in each patient. Much like their diagnostic findings, the findings they called “incidental” were exclusively among those 400–700 variants of potential health impact that they had previously selected through filters. Thus, even though the findings turned out not to be explanatory for the clinical condition of the patients in the end, they had been actively searched for and could have been expected from the study design. Hence, they did not occur incidentally and are research findings, or more precisely secondary findings according to the definition given above (health-relevant findings that were actively searched for although they were not the focus of the primary research question). The study by Yang et al. demonstrates that search strategies with long lists of variations of possible clinical relevance have huge potential to generate secondary health-related findings besides the intended diagnostic question. Hence, in the diagnostic setting, the ethical challenge of disclosure of secondary findings remains pertinent where broad search strategies are employed. However, usually in genetic diagnostics, the genomes or exomes of families are compared in a way that filters out heterozygote germline mutations. We are now seeing the first diagnostic exome and genome sequencing studies and it will be interesting to learn about the extent of such findings within future studies. It should be remembered, however, that diagnostic trials such as the one by Yang et al. are deeply embedded within the clinical context.

Thus, the common expectation that the compilation of ever-increasing genomic data sets would be paralleled by a similar increase in the numbers of IFs appears not to have been realized. In fact, current tools for handling big data sets avoid the detection of IFs. Owing to the quantity and complexity of genomic data (including epigenetic data), filtering tools become increasingly necessary. Therefore, it seems plausible that the unlikelihood of finding IFs in genomic research will not change in the future.

## Reframing the ethical discussion

The ongoing ethical and regulatory debate about IFs therefore needs to take into account the scarce evidence for IFs as well as the suggestion that filtering approaches make IFs unlikely. This reflects a general challenge for prospective bioethics: how to achieve the right timing and intensity of ethical and regulatory efforts for new technologies, particularly for biomedical applications. The difficulty of anticipating the ethical challenges arising from a new development can lead to a tough balance between uncertainty over the details and the need to shape the technology and its implementation [22]. Time, knowledge and power are critical dimensions in this debate and the challenging question is: “[W]hen to control? Early control might be possible due to the power to change situations and boundary conditions, but lacks knowledge about the consequences; late control can rely on much knowledge but is mainly powerless” [23].

One approach, used for the assessment of technological developments, is to tackle the difficulties caused by uncertainty by implementing procedures for monitoring and informing decision-makers about critical or unexpected changes, which allows people to act in time. We suggest applying a similar approach to bioethics. In order to have timely ethical solutions at hand it is important to develop solutions for plausible scenarios early on. In a second step, it is necessary to carefully evaluate whether the empirical projections that the theory is built on remain true. Then, the relevance of the scenario has to be substantiated or, if necessary, corrected. To date, the ethical debate about IFs has passed the first step. What we need is an evaluation and debate on the second step.

Assuming that our observations and reasoning concerning the occurrence of IFs are correct, the ethical discussion regarding IFs requires reframing. The first issue to be discussed is whether systematically avoiding IFs is ethically good or bad; the answer to this might differ depending on the research context or clinical field. One view is that neither researchers nor physicians in the clinical setting have a reason, let alone a duty, to maximize the likelihood of IF occurrence [24]. In that case, both should reduce the likelihood of IFs to a technically achievable minimum. Along these lines, the guidelines for diagnostic NGS published by the European platform EuroGentest favor targeted sequencing methods such as gene panels over genome-wide analysis in order to avoid IFs technically [25]. This policy has already been adopted by laboratories that use targeted NGS — that is, gene panels — instead of exome (or genome) sequencing, to pragmatically reduce the “hassle” of dealing with IFs.

The opposite view is that IFs have a potential health or reproductive importance for the study participant or patient: hence, if a researcher or a physician has a choice between two methods of genomic analysis, he or she should choose the one that allows for IFs (for example, by using whole-genome sequencing rather than somatic gene panels). This would be in line with those emphasizing the duties of genomic researchers towards research subjects [26, 27]. However, the practicability of the latter position as well as its potential impact on the occurrence of IFs are likely to be uncertain and conscribed.

Therefore, the ethical debate should shift to the important question of whether potential health-relevant variations should be targeted deliberately and systematically, for instance by the usage of positive lists and annotation algorithms, to benefit research participants and future patients. Here, the problem of unreliable and changing annotations of genetic variants and their medical significance in the scientific literature, as, for instance, pointed out by Rehm et al. [28] and Xue et al. [29], needs to be taken into consideration. In March 2013, in its above-mentioned paper, the ACMG issued the recommendation that laboratories performing clinical genetic sequencing seek and report back mutations as specified in their list of 56 pathogenic genes [16]. The authors also referred to this strategy by the term “opportunistic screening” as introduced by Wright et al. [30]. Amendola and colleagues extended the list to 112 genes and proved its large-scale feasibility [19]. However, usage of such lists raises several ethical questions, even if the idea of the obligatory return of findings to patients, as initially proposed by the ACMG, is excluded. Positive lists also provoke the question as to whether researchers have an obligation to actively search for health-relevant variants. Therefore, a careful evaluation of the arguments is necessary: the potential health benefits and potential psychological burdens for participants need to be balanced with the additional resources required for research projects with respect to time, effort and cost. While a duty for researchers to search for disease-causing variations seems far-fetched today, this might change in areas where the line between a diagnostic and research setting is getting increasingly blurred with the translation of genomic analyses from research to care [27]. Furthermore, the ethical and conceptual premises implied by any composition of positive lists need thorough analysis. It is certainly a challenge to decide upon the inclusion and exclusion criteria of such a positive list [31]. Criteria for determining the targets to be searched for, such as the benefit for patients and research subjects and the availability of treatments or preventive measures [16], raise questions concerning their precise definition and thresholds. Here, the ethical debate on IFs so far can make a valuable contribution, thanks to its consideration of the best practice for assessment and classification of findings [32, 33]. If we could agree on the content of such a list, its uptake should allow research participants and patients to benefit from new genome-scale sequencing techniques by being informed about possible findings from that kind of list. Since the term “incidental finding” would then be inappropriate [34], according to our terminology these findings would correctly be called “secondary findings”.

On the face of it, the use of a defined list of secondary findings appears to be a good thing, ethically speaking. The use of positive lists would supersede the discussion about IF-minimizing or IF-maximizing techniques and would allow patients and research subjects to be informed and prepared in advance for the kind of potential secondary findings that are on the list. Still, we would need to know whether the benefits yielded by testing for secondary findings outweigh potential burdens and costs for patients and research subjects as well as for research and the health system. In the context of research, this is important since any analysis beyond the original scope would have extra costs, possibly without benefit to the research project. These are questions that need to be answered empirically. Therefore, it would be desirable to monitor any uptake of the ACMG recommendation with regard to cost-effectiveness of the preventive measures.

In summary, the ethical debate on IFs in genomic research needs to be revisited. When NGS was first discussed, ethicists were forced to base their reflections upon hypothetical assumptions concerning IFs. The increase in genomic data made it plausible to project a parallel increase of IFs. To date, there is little evidence to support this hypothetical projection and therefore it needs to be carefully re-examined. At the moment, big sets of genomic data are handled with methods and filter techniques that avoid the occurrence of IFs in genomic research. In our opinion, this has not been taken sufficiently into account by those looking at the ethical implications of the field. A reframing of the ethical debate about IFs appears timely and appropriate.

## Abbreviations

ACMG: American College of Medical Genetics
DKFZ: German Cancer Research Center
EMBL: European Molecular Biology Laboratory
EURAT: Ethical and Legal Aspects of Whole Genome Sequencing
IF: Incidental finding
NCT: National Center for Tumor Diseases
NGS: next-generation sequencing
OMIM: Online Mendelian Inheritance in Man

## References

[1] Online Mendelian Inheritance in Man. http://www.omim.org.

[2] Wolf SM, Lawrenz FP, Nelson CA, Kahn JP, Cho MK, Clayton EW (2008). Managing incidental findings in human subjects research: analysis and recommendations. J Law Med Ethics.

[3] Christenhusz GM, Devriendt K, Dierickx K (2013). To tell or not to tell? A systematic review of ethical reflections on incidental findings arising in genetics contexts. Eur J Hum Genet.

[4] Zawati MH, Knoppers BM (2012). International normative perspectives on the return of individual research results and incidental findings in genomic biobanks. Genet Med.

[5] EURAT — Ethical and Legal Aspects of Whole Genome Sequencing. http://www.uni-heidelberg.de/totalsequenzierung/english.html.

[6] EURAT-Group (2013). Cornerstones for an ethically and legally informed practice of Whole Genome Sequencing.

[7] Bruno DL, Stark Z, Amor DJ, Burgess T, Butler K, Corrie S (2011). Extending the scope of diagnostic chromosome analysis: detection of single gene defects using high-resolution SNP microarrays. Hum Mutat.

[8] Kuan LC, Su MT, Kuo PL, Kuo TC (2013). Direct duplication of the Y chromosome with normal phenotype — incidental finding in two cases. Andrologia.

[9] Lewis A, James P (2012). An incidental finding of a large genomic deletion of BRCA1 on a molecular karyotype for a 5 year old child. Hered Cancer Clin Pract.

[10] Rostasy K, Fauth C, Gautsch K, Laimer I, Krabichler B, Wimmer K (2013). Modification of risk for cancer as a coincidental finding in DNA array investigation. Clin Genet.

[11] de Ligt J, Willemsen MH, van Bon BWM, Kleefstra T, Yntema HG, Kroes T (2012). Diagnostic exome sequencing in persons with severe intellectual disability. N Engl J Med.

[12] Williams JK, Daack-Hirsch S, Driessnack M, Downing N, Shinkunas L, Brandt D (2012). Researcher and institutional review board chair perspectives on incidental findings in genomic research. Genet Test Mol Biomarkers.

[13] Downing NR, Williams JK, Daack-Hirsch S, Driessnack M, Simon CM (2013). Genetics specialists’ perspectives on disclosure of genomic incidental findings in the clinical setting. Patient Educ Couns.

[14] Klitzman R, Appelbaum PS, Fyer A, Martinez J, Buquez B, Wynn J (2013). Researchers’ views on return of incidental genomic research results: qualitative and quantitative findings. Genet Med.

[15] Klitzman R, Buquez B, Appelbaum PS, Fyer A, Chung WK (2014). Processes and factors involved in decisions regarding return of incidental genomic findings in research. Genet Med.

[16] Green RC, Berg JS, Grody WW, Kalia SS, Korf BR, Martin CL (2013). ACMG recommendations for reporting of incidental findings in clinical exome and genome sequencing. Genet Med.

[17] Jurgens J, Ling H, Hetrick K, Pugh E, Schiettecatte F, Doheny K (2015). Assessment of incidental findings in 232 whole-exome sequences from the Baylor-Hopkins Center for Mendelian Genomics. Genet Med.

[18] Lawrence L, Sincan M, Markello T, Adams DR, Gill F, Godfrey R (2014). The implications of familial incidental findings from exome sequencing: the NIH Undiagnosed Diseases Program experience. Genet Med.

[19] Amendola LM, Dorschner MO, Robertson PD, Salama JS, Hart R, Shirts BH (2015). Actionable exomic incidental findings in 6503 participants: challenges of variant classification. Genome Res.

[20] Furtado CD, Aguirre DA, Sirlin CB, Dang D, Stamato SK, Lee P (2005). Whole-body CT screening: spectrum of findings and recommendations in 1192 patients. Radiology.

[21] Yang Y, Muzny DM, Reid JG, Bainbridge MN, Willis A, Ward PA (2013). Clinical whole-exome sequencing for the diagnosis of Mendelian disorders. N Engl J Med.

[22] Collingridge D (1980). The social control of technology.

[23] Liebert W, Schmidt J (2010). Collingridge’s dilemma and technoscience. Poiesis Prax.

[24] Christenhusz GM, Devriendt K, Vermeesch J, Dierickx K (2012). Why genomics shouldn’t get too personal: in favor of filters. Am J Med Genet A.

[25] EuroGentest Guidelines for Diagnostic Next Generation Sequencing. http://www.eurogentest.org/fileadmin/templates/eugt/pdf/NGS_Guidelines/EuroGentest_NGS_guidelines_2014_-_final_draft_02-12-2014_v2.pdf.

[26] Miller FG, Mello MM, Joffe S (2008). Incidental findings in human subjects research: what do investigators owe research participants?. J Law Med Ethics.

[27] Gliwa C, Berkman BE (2013). Do researchers have an obligation to actively look for genetic incidental findings?. Am J Bioethics.

[28] Rehm HL, Berg JS, Brooks LD, Bustamante CD, Evans JP, Landrum MJ (2015). ClinGen — The Clinical Genome Resource. N Engl J Med.

[29] Xue Y, Chen Y, Ayub Q, Huang N, Ball Edward V, Mort M (2012). Deleterious- and disease-allele prevalence in healthy individuals: insights from current predictions, mutation databases, and population-scale resequencing. Am J Hum Genet.

[30] Wright C, Burton H, Hall A, Moorthie S, Pokorska-Bocci A, Sagoo G (2011). Next steps in the sequence: the implications of whole genome sequencing for health in the UK.

[31] Burke W, Matheny Antommaria AH, Bennett R, Botkin J, Clayton EW, Henderson GE (2013). Recommendations for returning genomic incidental findings? We need to talk!. Genet Med.

[32] Wolf SM, Crock BN, Van Ness B, Lawrenz F, Kahn JP, Beskow LM (2012). Managing incidental findings and research results in genomic research involving biobanks & archived datasets. Genet Med.

[33] Berg JSKM, Evans JP (2011). Deploying whole genome sequencing in clinical practice and public health: meeting the challenge one bin at a time. Genet Med.

[34] Shkedi-Rafid S, Dheensa S, Crawford G, Fenwick A, Lucassen A (2014). Defining and managing incidental findings in genetic and genomic practice. J Med Genet.

